# Human Antimicrobial Peptide Isolated From *Triatoma infestans* Haemolymph, *Trypanosoma cruzi*-Transmitting Vector

**DOI:** 10.3389/fcimb.2018.00354

**Published:** 2018-10-30

**Authors:** Laura Cristina Lima Diniz, Antonio Miranda, Pedro Ismael da Silva Jr.

**Affiliations:** ^1^Special Laboratory of Toxinology, Butantan Institute, São Paulo, Brazil; ^2^Post-Graduation Program Interunits in Biotechnology, USP/IPT/IBU, São Paulo, Brazil; ^3^Department of Biophysics, UNIFESP, São Paulo, Brazil

**Keywords:** antimicrobial peptides, *Triatoma infestans*, fibrinopeptide A, innate immune system, internalization

## Abstract

The importance of antimicrobial peptides (AMPs) in relation to the survival of invertebrates is well known. The source and the mode of action on the insects' immune system of these molecules have been described from different perspectives. Insects produce their own AMPs as well as obtain these molecules from various sources, for example by absorption through the intestinal tract, as previously described for *Boophilus microplus*. Blood-sucking barber bug *Triatoma infestans* attracts social, economic and medical interest owing to its role in the transmission of Chagas disease. Despite new studies, descriptions of AMPs from this insect have remained elusive. Thus, the aims of this work were to characterize the antimicrobial potential of human fibrinopeptide A (FbPA) obtained from the *T. infestans* haemolymph and identify its natural source. Therefore, FbPA was isolated from the *T. infestans* haemolymph through liquid chromatography and identified by mass spectrometry. This peptide exhibited antimicrobial activity against *Micrococcus luteus*. Native FbPA from human blood and the synthetic FbPA also exhibited antimicrobial activity. The synthetic FbPA was conjugated with fluorescein isothiocyanate and offered to the insects. The haemolymph collected after 72 h exhibited fluorescence at the same wavelength as fluorescein isothiocyanate. Our experiments show that beyond intrinsic AMP production, *T. infestans* is able to co-opt molecules via internalization and may use them as AMPs for protection.

## Introduction

The discovery of insect fossils that are approximately 400 million years old, such as those of *Rhyniella praecursor* and *Rhyniognatha hirsti*, demonstrates that insects were among the first animals to conquer the terrestrial habitat (Scourfield, 1940; Ross, 2017). Their evolution represents a successful process represented by their earth-wide distribution, inhabiting every environment except marine habitats.

These evolutionary achievements are mainly due to some basic defense lines which have evolved over time. The immune system of these animals is frequently a subject of study and well described, whereas the adaptive immunity still relies on theories and only some molecules have been described (Kurtz and Franz, 2003; Little et al., 2005; Watson et al., 2005; Sadd and Schimdt-Hempel, 2006).

The innate immune system is divided into cellular and humoral responses that act together in three interconnected cascades (Hoffman et al., 1996). The first is composed of enzymatic activation, the second is the fast and temporary production of antimicrobial peptides (AMPs), both belonging to the humoral response, and the third refers to phagocytic and encapsulation defenses, mediated specially by haemocytes (Hoffmann, 1995; Strand and Pech, 1995; Gillespie et al., 1997; Blandin and Levashina, 2004; Cerenius and Soderhall, 2004; Theopold et al., 2004; Irving et al., 2005; Strand, 2008; Pasupuleti et al., 2012).

Among the soluble molecules related to the humoral immunity, a growing interest in the AMP class emerged as several molecules with unique properties were discovered (Stephens, 1962), such as cecropins (Steiner et al., 2009) and defensins (Ganz et al., 1985). AMPs are generally amphipathic and cationic and have high hydrophobic properties at physiological pH. Due to these physical characteristics, AMPs are more likely to form alpha helixes. This property may help with peptide penetration and disruption of negatively charged microbial membranes. As this is a charge-based interaction, AMPs may act in an independent protein-binding manner (De Simone and Souza, 2000), which makes them more likely to evade resistance mechanisms. The specificity of AMPs toward target cell membranes correlates not only with prokaryotic membrane composition but also with the topological arrangement of their lipids (Matsuzaki, 1999; Pushpanathan et al., 2013). Bacterial membranes contain large amounts of negatively charged phospholipids, whereas eukaryotic cells, specifically mammalian cell membranes, are composed almost exclusively of electrically neutral lipids (Matsuzaki, 1999). These features suggest why AMPs tend to be less toxic to eukaryotic cells.

Recent studies have demonstrated an increase in antimicrobial drug resistance. In 2000, bacteria resistant to one or more antibiotic classes started being described more commonly (Tavares, 2000; Santos Filho et al., 2002; Figueiredo et al., 2009; Zanol et al., 2010; Neves et al., 2011). The increasing rate of resistance and the diversity of resistant bacteria are two of the main justifications for research into the production of new antimicrobial drugs (Ferreira et al., 2001; Santos, 2004; Brito and Cordeiro, 2012).

The AMPs represent an interesting alternative to commercial antibiotics due to factors such as their low toxicity to eukaryotic cells, specificity for bacterial cell membranes, potential antifungal, antiparasitic, and antitumour activities, impact on cell differentiation, as well as vasculogenesis, antiobesity, antiviral, wound healing, and cell recruitment properties (Liang and Kim, 1999; Yi et al., 2014; Mahlapuu et al., 2016; Marxer et al., 2016; Mylonakis et al., 2016; Tonk et al., 2016).

Owing to their medical relevance in South and Central America, triatomine insects represent the main target of several fields of research ranging from public health to host–pathogen evolution. However, few studies have shown the role of AMPs in triatomine–pathogen interactions.

The isolation of the prolixin AMP from *Rhodnius prolixus*, expression of three different defensins by *Pyrrhocoris apterus* (Cociancich et al., 1993), expression of two defensins (def3 and def4) in several tissues of the barber bug *Triatoma brasiliensis* (Waniek et al., 2009), a description of trialysin expression in the salivary glands of *Triatoma infestans* (Assumpção et al., 2008) and two different types of digestive tract lysozymes (Kollien et al., 2003; Balczun et al., 2008; Flores-Villegas et al., 2015) provide evidence for the role of AMPs in triatomine immune defense mechanisms.

Although there is evidence of AMP production by triatomines, there are no published descriptions of antimicrobial molecules isolated from *T. infestans* haemolymph yet. Four AMPs were characterized among ten isolated from *T. infestans* blood (Diniz, 2016—unpublished data). The most relevant isolated finding was the presence of human fibrinopeptide A (FbPA) with antimicrobial activity.

Regarding the relevance of the description of AMPs, elucidation of their role in the invertebrate immune system and, consequently, development of new AMP-dependent drugs, our aim was to identify and determine the origin of AMPs isolated from the Chagas disease-transmitting vector *T. infestans* haemolymph. By combining mass spectrometry approaches with functional assays, our results provide evidence that *T. infestans* is able to assimilate molecules through feeding and use them as part of their immune system, probably functioning as AMPs circulating in the haemolymph.

## Methods

The experiments were performed under the exemption of the *Animal Research Ethics Committee* (CEUAIB—Comitê de ética no uso de animais do Instituto Butantan) n° I-1345/15.

### Bacterial strains

The microorganisms *Micrococcus luteus* (strain A270), *Staphylococcus aureus* (ATCC 29213), *M. luteus* (Nalidixic resistant), *Bacillus megaterium* (ATCC 10778), *Bacillus subtilis* (ATCC 6633), *Escherichia coli* (SBS363), *Enterobacter cloacae* β-12, *Alcaligenes faecalis* (ATCC 8750), *Serratia marcescens* (ATCC 4112), *Pseudomonas aeruginosa* (ATCC 27853), *Candida parapsilosis* (IOC 4564), *Candida albicans* (IOC 4558), *Cryptococcus neoformans, Saccharomyces cerevisiae, Candida tropicalis* (IOC 4560), *Cladosporium* sp. (bread isolated), *Penicillium expansum* (bread isolated), *Aspergillus niger* (bread isolated), *Paecilomyces farinosus* (IBCB-215), and *Cladosporium herbarum* (ATCC 26362) were obtained from the Special Laboratory of Toxinology, Butantan Institute (São Paulo, Brazil).

### Animals

*Triatoma infestans* were obtained from the Ecolyzer Group Entomology Laboratory and kept alive in the vivarium of the Special Laboratory of Toxinology, Butantan Institute (São Paulo, Brazil) at 37°C and fed every 2 weeks with human blood from a healthy volunteer donor, in the presence of citrate buffer (150 mM, pH 7,4) (Martins et al., 2001).

### Bacteria inoculation and haemolymph collection

One week after blood feeding, adult *T. infestans* were injured with needles soaked in an *E. cloacae* and *M. luteus* pool, both at logarithmic-phase growth. After 72 h, 300 μL of haemolymph was collected by excising the metathoracic legs and pressing on the abdomen of the *T. infestans* (Boman et al., 1974) in the presence of phenylthiourea (PTU), to avoid the activation of the phenoloxidase cascade, and stored at −80°C until use.

### Sample fractionation

#### Acid and solid-phase extractions

To release the contents of the haemocytes, the sample was incubated in acetic acid (2 M) for 5 min and centrifuged at 16.000 × g for 30 min at 4°C. The supernatant was injected into coupled Sep-Pack C_18_ cartridges (Waters Associates) equilibrated in 0.1% trifluoroacetic acid (TFA). The sample was eluted in three different acetonitrile (ACN) concentrations (5, 40, and 80%) and then concentrated and reconstituted in ultrapure water.

#### Reverse-phase high-performance liquid chromatography (RP-HPLC)

RP-HPLC separation was performed with a C_18_ column (Jupiter, 10 × 250 mm) equilibrated with 0.05% TFA. The elution gradient for the 5% ACN fraction was 2% to 20% (v/v) of solution B (0.10% (v/v) TFA in ACN) in solution A (0.05% (v/v) TFA in water). For the 40% ACN fraction, the gradient was 2–60% of solution B in solution A, and for the 80% ACN fraction, the gradient was 20–80% of solution B in solution A.

RP-HPLC was performed for 60 min at a 1.5 mL/min flow rate. Effluent absorbance was monitored at 225 nm, and the fractions corresponding to absorbance peaks were hand-collected, concentrated under vacuum, and reconstituted in ultrapure water.

When necessary, a second chromatographic step was performed on a VP-ODS analytic column (Shim-pack®), with a 1.0 mL/min flow rate for 60 min. This was performed to guarantee sample homogeneity. The gradients for these second chromatographic stages were determined by the target molecule's retention time.

### Liquid growth inhibition assay

The antimicrobial assay was performed against all the microorganisms listed previously in Methods section Bacterial Strains, using poor broth nutrient medium (PB: 1.0 g peptone in 100 mL of water containing 86 mM NaCl at pH 7.4; 217 mOsM) and Müller-Hinton medium (peptone 5.0 g/L; casein peptone 17.5 g/L; agar 15.0 g/L; Ca^2+^ 20.0–25.0 mg/L; Mg^2+^ 10.0–14.5 mg/L; pH 7.4) for bacteria and potato dextrose broth (1/2 PDB: 1.2 g potato dextrose in 100 mL of H_2_O at pH 5.0; 79 mOsM), and RPMI 1640 (Roswell Park Memorial Institute medium) medium with MOPS 0.165 mol/L [RPMI without bicarbonate 10.4 g/L; MOPS (3-(n-morpholino) propanesulphonic acid) 34.53 g/L; pH 7.0] at half-strength for fungi (Bulet et al., 1993; Wayne, 2008).

Antimicrobial activity was determined using a five-fold microlitre broth dilution assay in 96-well sterile plates at a final volume of 100 μL. A mid-log-phase culture was diluted to a final concentration of 1 × 10^5^ colony-forming units/mL. The dried fractions were dissolved in 500 μL of ultrapure water, and 20 μL of this was added to each well. We then added 80 μL of microorganism dilution. To determine the minimal inhibition concentration (MIC), the bacterial growth rates were measured after an 18 h incubation. To determine the minimal bactericidal concentration (MBC), the bacterial growth rates were measured after 96 h at 595 nm (Hancock, 1999; Yamamoto, 2003).

### Mass spectrometry (LC/MS)

Mass spectrometry analysis was performed on an LTQ XL (Thermo Scientific). The equipment was previously calibrated with the following substances: caffeine (m/z 194.5), L-MRFA acetate in water (m/z 524.3), and Ultramark 1621. Ovalbumin was used as molecular weight control (43 kDa). The samples were concentrated and diluted in 15 μL 0.1% formic acid (FA). For the liquid chromatography, a C_18_ column (Waters) was used with an ACN gradient linear from 0 to 80% in acidic water (FA 0.1%) during 60 min at a 400 nL/min flow. The spectrometer was set to a positive parameter.

### Computational analysis

Mass spectrometry data were analyzed with Xcalibur 5.0 (Thermo Electron, EUA) and Mascot Deamon® version 5.4.2, using Swiss-Prot and NCBInr Insects, Hemipteran, Triatomines and Fibrinogen banks for database comparison. The homology searches for possible results were performed on the following databases: ArachnoServer Spider Toxin Database www.arachnoserver.org; The Arthropoda PartiGeneDatabases www.nematodes.org/NeglectedGenomes/ARTHROPODA; PepBank pepbank.mgh.harvard.edu; Vector Base pepbank.mgh.harvard.edu; APD2: Antimicrobial Peptide Calculator and Predictor and BLAST (NCBI) aps.unmc.edu/AP/main.html.

The data were also analyzed through PEAKS® (Bioinformatics Solutions Inc.) with Insects, Hemipteran, Triatomines, and Fibrinogen databases obtained on UniProt (www.uniprot.org, 1243446; 133071; 31334; and 12342 sequences, respectively, March 25th, 2015). The results were considered valid only when they were reproducible in a different analysis.

### Solid-phase peptide synthesis

Peptides were synthesized by the solid-phase method (Miranda et al., 1994), using a methylbenzhydrylamine resin (MBHAR) and employing the *t*-Boc strategy. After cleaving the peptides from the resin, peptides were purified from the lyophilized crude solutions by HPLC on a C_18_ column. To guarantee high purity and to characterize the peptides, LC-ESI-MS equipment was used.

### Synthetic peptide concentration

Peptide concentrations were determined by using the Lambert–Beer law using the molar extinction coefficient at 205 nm absorption (Anthis and Clore, 2013), obtained using the tool available at http://nickanthis.com/tools/a205.html.

### Internalization assays

#### Preparation of fluorescein conjugate

Fluorescein isothiocyanate (FITC) isomer I (Sigma-Aldrich®) was used by following the protocol provided by Sigma-Aldrich®. FITC was dissolved in dry DMSO at a concentration of 1 mg/mL and protected from light. For coupling, 150 μL of FITC solution was added, 5 μl at a time, to the synthetic FbPA solution (2 mM) and kept for more than 8 h at 4°C in the dark. Ammonium chloride was added to a final concentration of 50 mM and incubated for 2 h to quench the reaction. The FITC–FbPA conjugate was then purified [Methods section: Acid and Solid Phase Extractions and Reverse Phase High-Performance Liquid Chromatography (RP-HPLC)].

#### Blood feeding and haemolymph extraction containing the fluorescein conjugate

After the purification, 3 mg of the conjugated FbPA were diluted in 4 mL human blood and offered to the insects (Martins et al., 2001). The haemolymph from the engorged animals without bacterial challenge was collected 72 h after the blood feeding and then submitted to fluorescence measurement and purification [Methods section: Acid and Solid Phase Extractions and Reverse Phase High-Performance Liquid Chromatography (RP-HPLC)]. The haemolymph of non-fed animals was also collected for fluorescence control comparisons.

### Human fibrinopeptide a isolation

Human blood was extracted to a final volume of 10 mL and incubated at 37°C until complete clot formation. The clot and plasma obtained were macerated in the presence of PBS and centrifuged at 16,000 × g for 5 min. The supernatant was filtered with PVDF membrane filters (Merck Millipore®, 25 mm; 0.45 μm) and then purified [Methods section: Acid and Solid Phase Extractions and Reverse Phase High-Performance Liquid Chromatography (RP-HPLC)]. The fraction corresponding to the human FbPA was isolated and submitted for antimicrobial assay (Methods section: Liquid Growth Inhibition Assay).

### Fluorescence measurements

The fluorescence evaluation was performed in a Perkin Elmer® Wallac 1420 VICTOR 2™ as triplicates nine times within a 15-min gap between them. The statistical analyses of variances were performed through the one-way analysis of variance (ANOVA) (single variant); results were significant when *p* < 0.05.

When necessary, Thermo Scientific NanoDrop™ fluorescence measurements were performed. Both readings used a 495 nm filter for excitation and a 520–530 nm filter for emission.

## Results

### Purification/isolation

**Figure 1:**
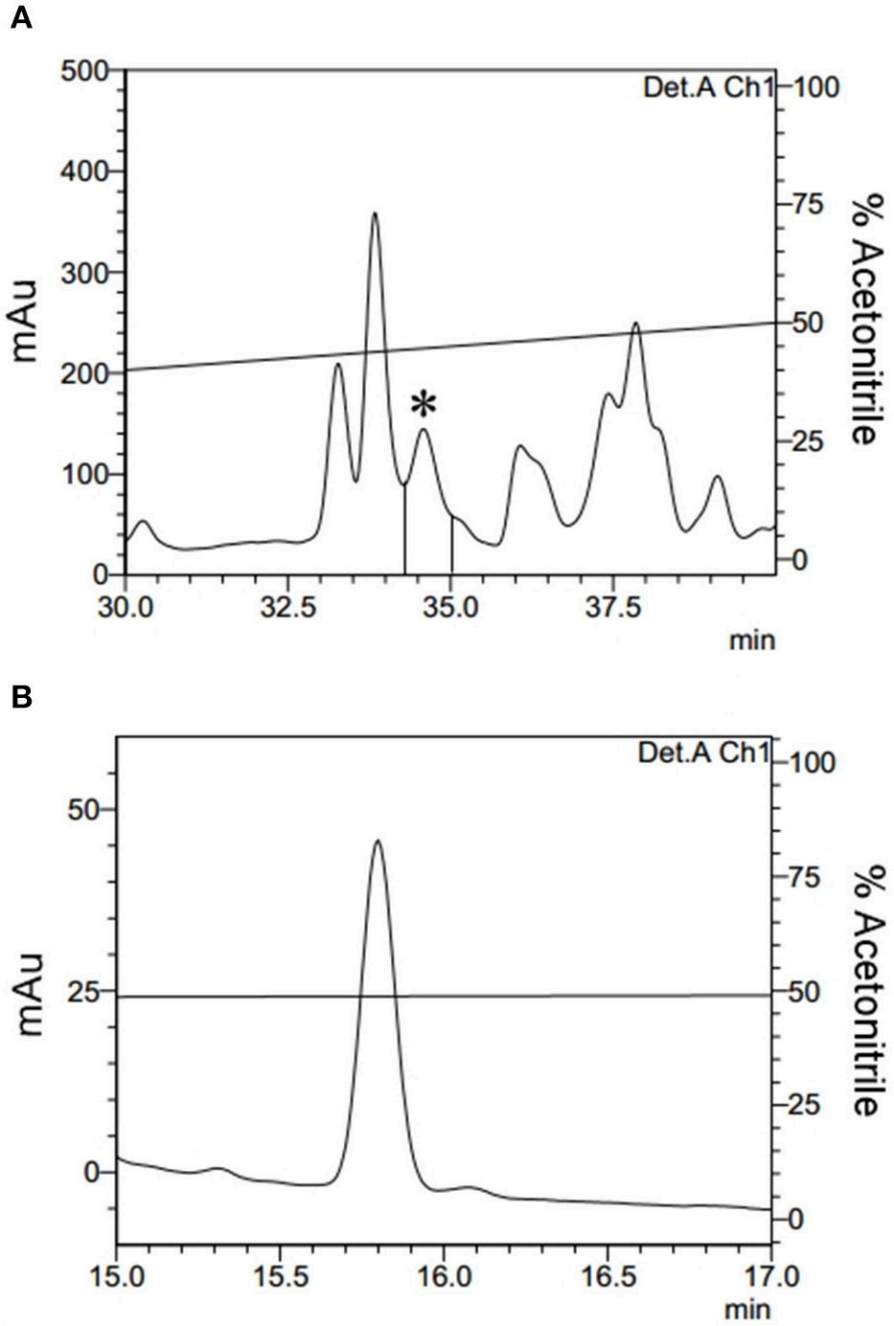
Isolation of Fibrinopeptide A. **(A)** The 80% ACN fraction isolated from *Triatoma infestans* haemolymph was separated by RP-HPLC using a C_18_ column, eluted with a linear gradient from solution A from 20 to 80% of the solution B run for 60 min. The labeled fraction (*), eluted at 34.4 min, exhibited antimicrobial activity and was submitted to a second chromatography step. **(B)** The second RP-chromatographic step on an analytic VP-ODS column, with an ACN gradient from 47 to 57% solution B in 60 min, to guarantee its homogeneity.

Acid was extracted from the total haemolymph and, subsequently, via three sequential ACN elutions leading to the separation of the main sample into three different fractions according to the ACN concentration (Methods section: Acid and Solid Phase Extractions). During the 80% ACN fraction purification via HPLC, a fraction eluted at 34.4 min. A fraction eluted at 34.4 min demonstrated antimicrobial activity and was completely isolated (Figures 1A,B).

**Figure 2:**
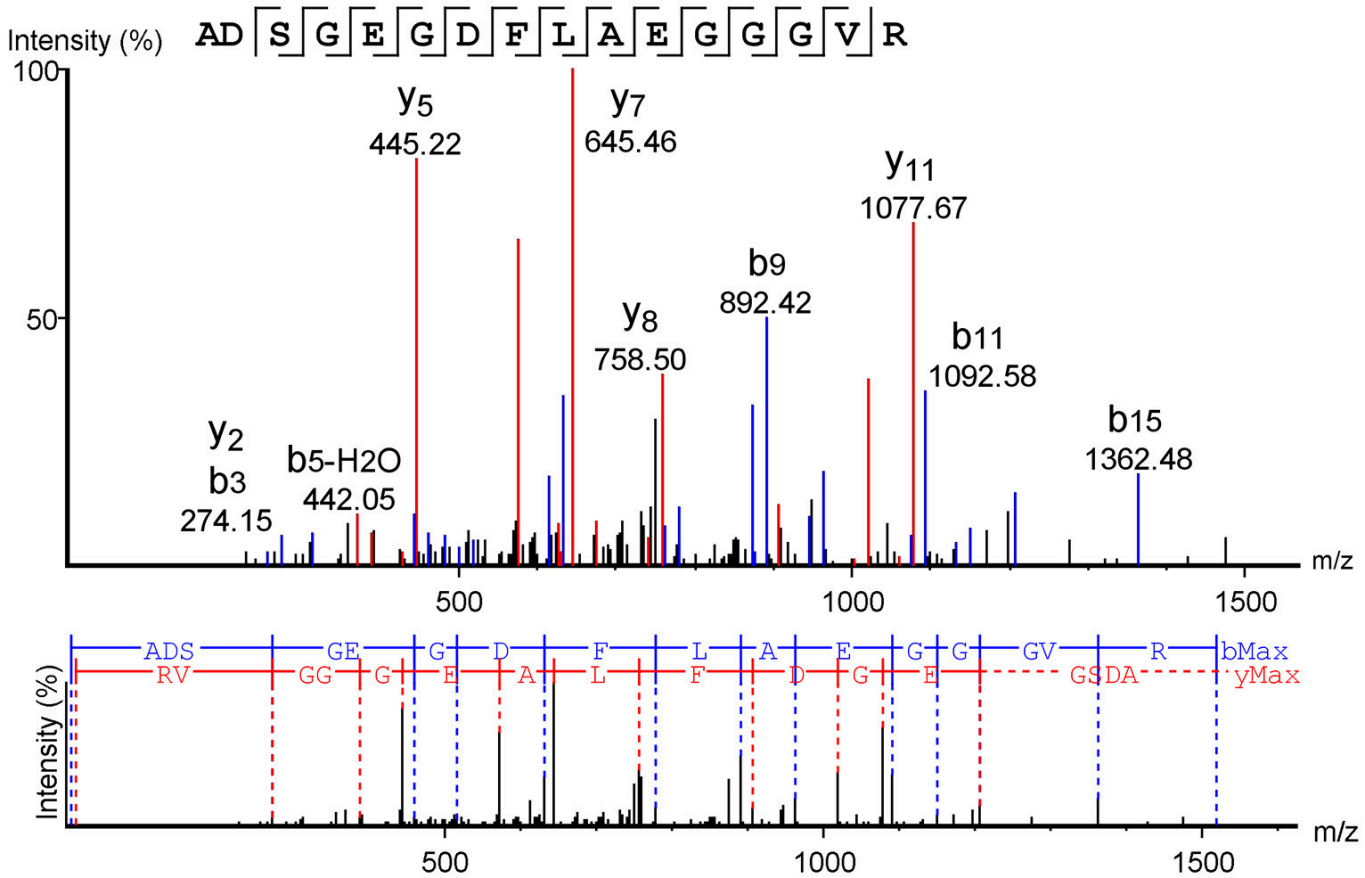
Fibrinopeptide A mass spectrometry analysis. Mass spectrometry data analysis from the software Peaks™, using the Swiss-Prot database as a comparison. The y-series is represented in red, and the b-series in blue.

When analyzed using the MASCOT® software to search the Swiss-Prot database, mass spectrometry data from the isolated molecule showed high identity with the human fibrinogen alpha chain. The sequence obtained from Peaks software confirmed this result, demonstrating that the sequence obtained corresponds to the human FbPA located on the N-terminal portion of the alpha chain of human fibrinogen (Figure 2).

**Figure 3:**
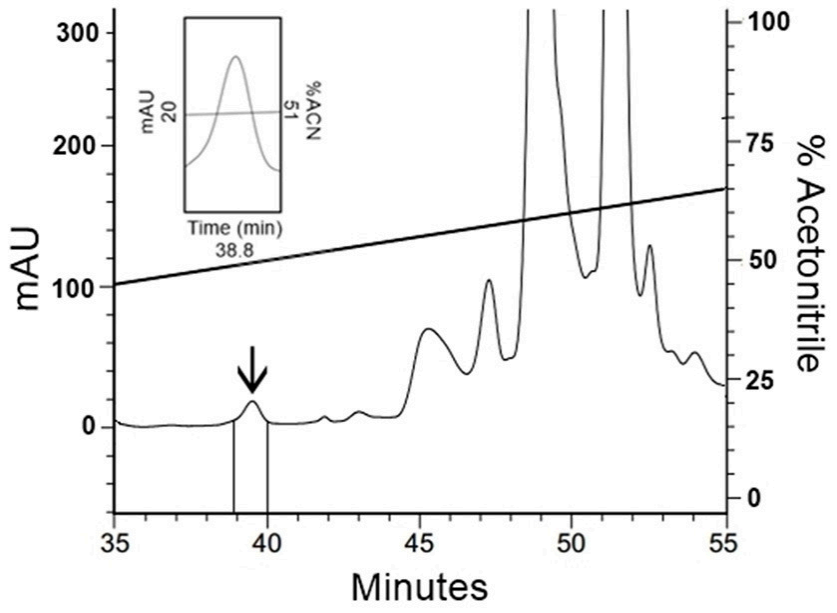
Isolation of human Fibrinopeptide A. The extract from coagulated human blood was separated by RP-HPLC using a C_18_ column, eluted with a linear gradient from solution A from 20 to 80% on solution B run for 60 min. Expanding the chromatogram, it is possible to see the fraction labeled with an arrow, eluted at 38.8 min, that corresponds to FbPA.

To confirm the results obtained with native human FbPA, human blood was processed (Methods section: Human Fibrinopeptide A Isolation) and the target fraction was isolated by HPLC (Figure 3); this fraction also exhibited antimicrobial activity. Mass spectrometry data analysis from the isolated fraction (not shown) confirms the molecular weight present in the fraction to be equivalent to that expected for human FbPA, suggesting that the antimicrobial results are due to FbPA enrichment in this fraction.

### Antimicrobial activity

The major antimicrobial activity of the human FbPA isolated from the *T. infestans* haemolymph when tested via the liquid growth inhibition assay was against *M. luteus* (A270) at 0.002–0.005 mg/mL concentrations. Due to the small amount of the native FbPA isolated from human blood, it was tested directly against *M. luteus* and exhibited activity in the same concentration range.

FbPA was synthetized to supply the amount of native sample obtained from the HPLC purifications. As a matter of comparison, the synthetic FbPA and the FITC–FbPA conjugate were also tested against *M. luteus* (A270); the first was active at 0.06–0.12 mg/mL and the second at 0.01–0.02 mg/mL (Table 1), indicating a decrease on the antimicrobial potential of the synthetic molecules.

**Table 1:** Antimicrobial activity concentrations.

**Microorganism**	***Micrococcus luteus*** **A270**			
FbPA source	*T. infestans*' haemolymph	Human blood	S-FbPA	S-FbPA+FITC
Concentration (mg/mL)	0.002–0.005	0.002–0.005	0.04–0.08	0.01–0.02

*Minimal interval concentration required for natural and synthetic molecules to exhibit activity against Micrococcus luteus A270. S-FbPA, the synthetic peptide; S-FbPA+FITC, the synthetic peptide–fluorescein conjugate*.

The synthetized FbPA was tested with a broad range of bacterial and fungal species and showed antimicrobial activity against *Pseudomonas aeruginosa, Escherichia coli, Candida parapsilosis, Cryptococcus neoformans, Candida tropicalis, Paecilomyces farinosus, Cladosporium* sp., and *Penicillium expansum*. Only those susceptible to FbPA's antimicrobial activity are listed in the table below (Table 2).

**Table 2:** Synthetic fibrinopeptide A's antimicrobial activities against bacterial and fungal strains.

**Microorganism**	**MIC**		**MBC**	
	μ**M (mg/mL)**		μ**M (mg/mL)**	
**MEDIA**	**MH**	**PB**	**MH**	**PB**
**GRAM-POSITIVE BACTERIA**				
*Micrococcus luteus*	NA^a^	42–84 (0.04–0.08)	NA	NA
**GRAM-NEGATIVE BACTERIA**				
*Pseudomonas aeruginosa*	NA	5.2–10.5 (0.005–0.01)	NA	NA
*Escherichia coli*	NA	5.2–10.5 (0.005–0.01)	NA	NA
**MEDIA**	**RPMI**	**PDB**	**RPMI**	**PDB**
**EASTS**				
*Candida parapsilosis*	NA	42–84 (0.04–0.08)	NA	NA
*Cryptococcus neoformans*	NA	42–84 (0.04–0.08)	NA	NA
*Candida tropicalis*	NA	5.2–10.5 (0.005–0.01)	NA	5.2–10.5 (0.005–0.01)
**FILAMENTOUS FUNGI**				
*Cladosporum sp*.	42–84 (0.04–0.08)	42–84 (0.04–0.08)	NA	42–84 (0.04–0.08)
*Penicilium expansum*	42–84 (0.04–0.08)	42–84 (0.04–0.08)	NA	42–84 (0.04–0.08)
*Paecilomyces farinosus*	NA	42–84 (0.04–0.08)	NA	42–84 (0.04–0.08)

Minimal inhibition concentration (MIC) and minimal bactericidal concentration (MBC) values obtained on the liquid growth inhibition assay. NA, not active on a concentration of 84 μM. PB, poor broth nutrient medium; MH, Müller-Hinton medium. PDB, potato dextrose broth medium; and RPMI, Roswell Park Memorial Institute medium

The synthetic FbPA concentrations effective as an antimicrobial varied according to the microorganism class. The main bacteriostatic activity was against *P. aeruginosa* and *E. coli*, at 0.005–0.01 mg/mL in the PDB medium. The main fungicidal activity was against *Cladosporium* sp. and *P. expansum*, both at 0.06–0.12 mg/mL n the RPMI medium and C. tropicalis at 0.005–0.01 mg/mL in PDB medium. These results prove that FbPA has a potent and effective antimicrobial activity against different microorganisms.

### Internalization assays

After confirming that the isolated molecule had 100% homology to the human FbPA and confirming that all of the isolated and produced FbPA had similar antimicrobial activity, our next step was to verify the origin of the molecule isolated from the *T. infestans* haemolymph.

To carry out this, the synthetic FbPA was coupled to a fluorescent probe (FITC) and the insects were fed with blood containing incremental amounts of the FITC–FbPA complex.

#### FITC–FbPA coupling confirmation

First, to verify the FITC–FbPA coupling and its fluorescence, the complex was examined via NanoDrop™ scan. The conjugate presents one FITC molecule for each FbPA sequence, confirming the coupling (data not shown). The fluorescence scan shows that the complex has a significant emission.

#### Internalization

To confirm the hypothesis of FbPA internalization by the *T. infestans*, the insects were fed with human blood containing the fluorescent FITC–FbPA conjugate and their haemolymph was collected and analyzed.

**Figure 4:**
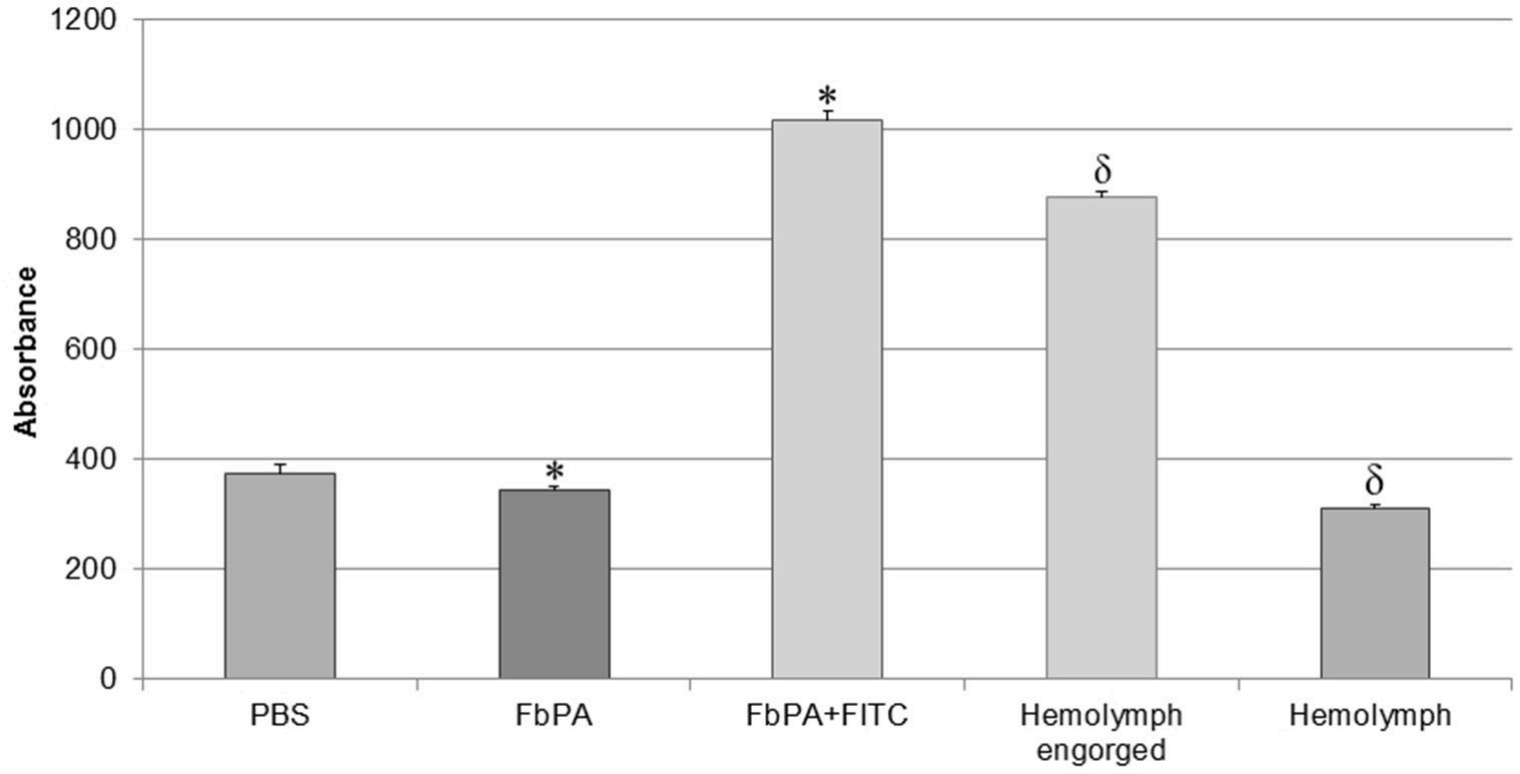
Comparison of haemolymph fluorescences. Comparison of the haemolymphs of engorged and non-fed insects. Through excitation with 495 nm, it is possible to observe the significant difference between the synthetic FbPA and the FITC–FbPA conjugate (*p = 3.84^−17^), as previously shown. The haemolymph of the engorged insects also has a significant difference when compared to the haemolymph of non-fed insects (δ*p* = 1.7^−21^). The fluorescence evaluation was carried out in a Perkin Elmer® Wallac 1420 VICTOR 2™, as triplicates and at nine times within a 15-min gap between them. Statistical evaluations were made with the ANOVA (single variant).

After feeding the insects with blood containing the FITC–FbPA conjugate, the haemolymph was collected from both engorged and non-fed insects for general fluorescence evaluation, verifying if the insect was able to absorb the fluorescently labeled peptide (Figure 4).

We observed the expected difference between the haemolymphs: the engorged insect's haemolymph exhibits a higher absorbance, comparable to the positive control (FITC–FbPA conjugate), while the non-fed insect's haemolymph showed no absorbance. This demonstrates that at least part of the conjugate was absorbed by the insects. Based on this, the haemolymph with fluorescence was tested by HPLC as previously described [Methods section: Reverse Phase High-Performance Liquid Chromatography (RP-HPLC)] to isolate the internalized conjugate.

**Figure 5:**
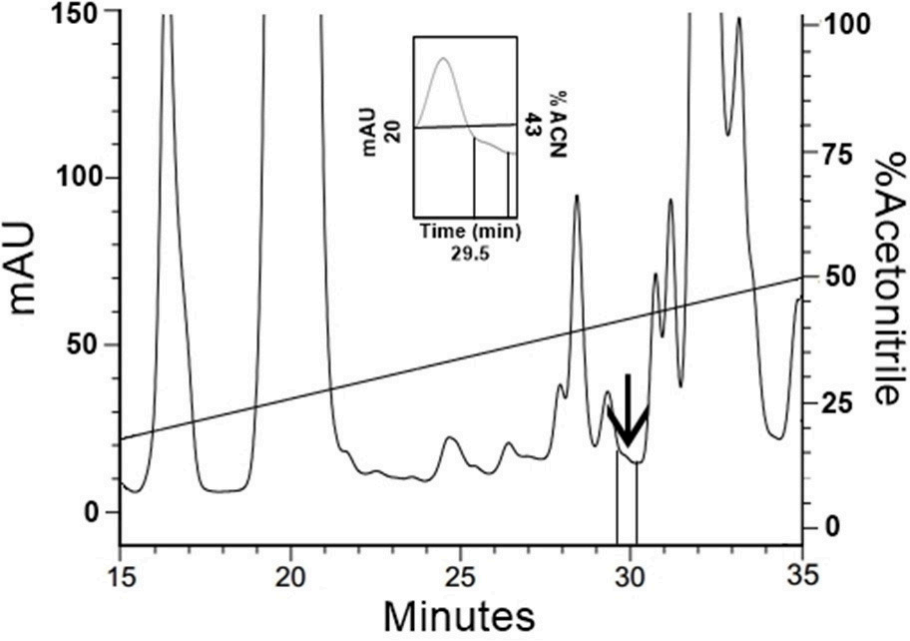
Isolation of FITC–FbPA conjugate on the insects haemolymph. The haemolymph from the engorged insects was separated by RP-HPLC using a C_18_ column, eluted in a linear solution A gradient from 20 to 80% on solution B for 60 min. Zooming the chromatogram, it is possible to see the fraction, eluted at 29.5 min in 43% ACN, that corresponds to the conjugate.

Based on the fact that the initial FbPA isolated from the *T. infestans* haemolymph was eluted in 43% ACN, the fractions eluted between 40 and 50% ACN had their fluorescence evaluated. The fraction eluted with 43% ACN exhibited the predicted fluorescence emission (Figure 5). The mass spectrometry data of this fraction confirms that the material from the isolated fraction corresponds to the FITC–FbPA conjugate (data not shown).

**Figure 6:**
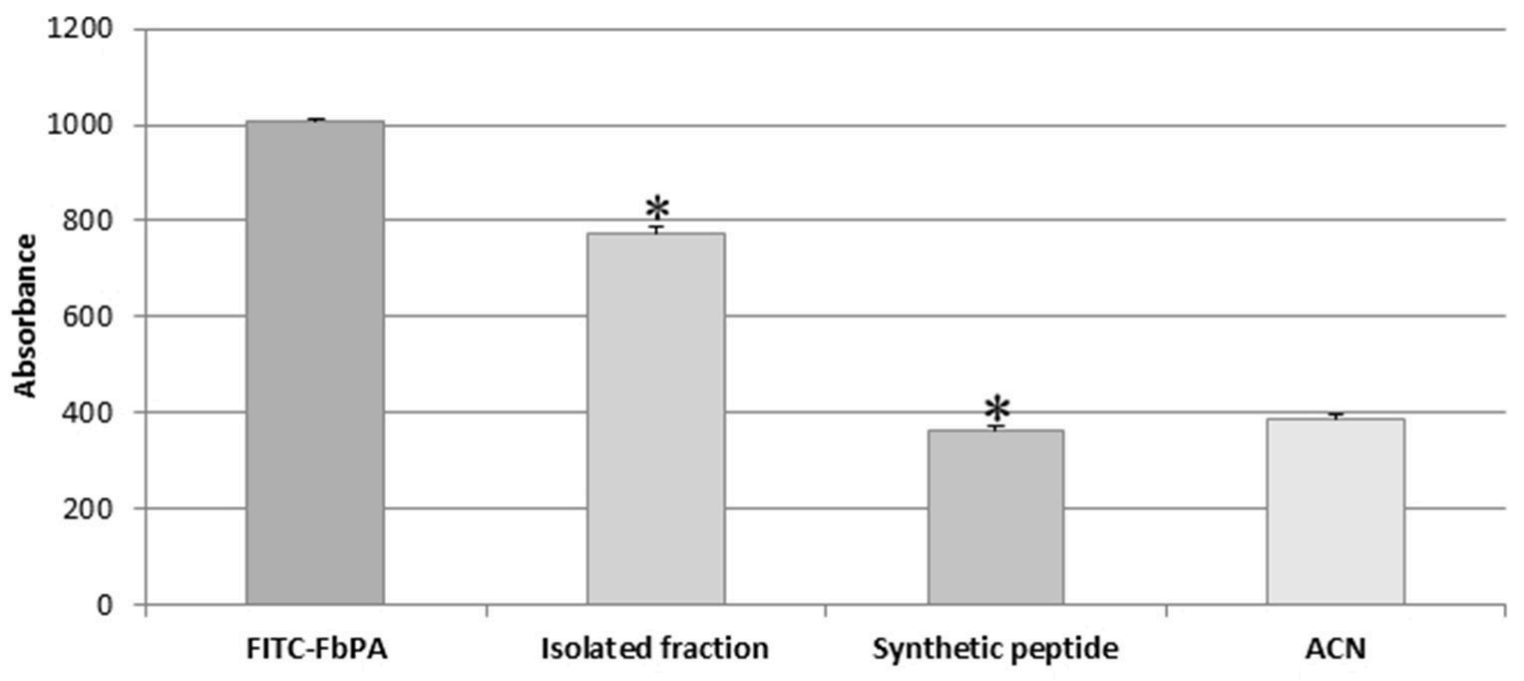
Isolated fraction fluorescence evaluation. Determination of the difference on the emitted fluorescence between the internalized fraction (FITC–FbPA conjugate) and the synthetic FbPA (*p = 1.85^−15^). The evaluation was performed in a Perkin Elmer® Wallac 1420 VICTOR 2™, as triplicates and nine times within a 15-min gap between them. Statistical evaluations were made with the ANOVA (single variant).

Fluorescence readings were taken via excitation with a 495 nm filter and a 520–530 nm emission filter as a method to evaluate and compare the fluorescence emissions from the isolated fraction, the FITC–FbPA conjugate, and by the synthetic FbPA (Figure 6). The isolated fraction was tested via the inhibition assay (Methods section: Liquid Growth Inhibition Assay) and was active against *M. luteus* at 0.02 mg/mL.

## Discussion

Although several studies have been performed aimed at a wider comprehension of the immune system of invertebrates, there are no consistent data about their adaptive immunity. It is known that there is no cell-based immunological memory, but some molecules produced by invertebrates have been described, such as molecules related to the immunoglobulin G superfamily (Watson et al., 2005). Other studies were able to demonstrate a certain specificity of the immune response in a second pathogen exposure (Kurtz and Franz, 2003; Little et al., 2005; Sadd and Schimdt-Hempel, 2006).

As insects do not have a relevant immune memory, a rapid response mechanism is required. Therefore, AMPs play an important role on the immunological response of these animals.

Commonly, AMP levels in the insect's haemolymph without bacterial or fungal contamination is low, increasing only with the stimuli of invasion/infection. As insects lack immunological memory, there is the necessity to activate intracellular pathways to induce the production of AMPs against each microorganism invasion. The main AMP production pathways activated are the *Spaetzle-Toll*—activated by fungi and gram-positive bacteria—and *Imd*—activated by gram-negative bacteria (Schmid-Hempel, 2005). Representing rapid responses, the peptide production does not exceed 8 h (Dunn, 1986). Confirming that FbPA has antimicrobial activity represents a huge step because it reinforces the idea of evolutionary improvements of the *T. infestans* immune system.

FbPA was obtained from the *T. infestans* haemolymph in the presence or absence of bacterial challenge and was active against several microorganisms in both cases. Thus it is possible to infer that the presence of this molecule in the insect's haemolymph is relevant to the insect's protection, whereas it is a known fact that AMPs can act in synergy and potentialize their effect over a target cell (Aaron et al., 2000; Yan and Hancock, 2001; Pamma et al., 2012; Doern, 2014; Nuding et al., 2014; Zerweck et al., 2017). It is still unknown whether *T. infestans* internalizes other molecules from the human blood. Diniz (2016) isolated ten AMPs and identified four among them, but unlike FbPA, none of the AMPs described belonged to the blood ingested.

Few studies have been performed to identify the antimicrobial activities of fibrinopeptides. Although the work performed by Påhlman et al. (2013) was unable to prove FbPA's antimicrobial action, Tang et al. (2002) demonstrated that seven molecules derived from human platelets (including fibrinopeptides A and B) exhibited antimicrobial activity. The authors also confirmed that FbPA exhibited antimicrobial activity against *E. coli, S. aureus, C. albicans*, and *C. neoformans*, while our work demonstrated its activity against *M. luteus, P. aeruginosa, E. coli, C. parapsilosis, C. neoformans, C. tropicalis, P. farinosus, Cladosporium sp*., and *P. expansum*.

The native FbPA isolated has activity at a 0.002–0.005 mg/mL concentration, while the synthetic FbPA has activity at a 0.1–0.2 mg/mL concentration. This discrepancy can be explained by the fact that the synthetic peptide has an amide group in its C-terminal portion, whereas the original sequence has a carboxyl group in the C-terminal portion. This change to the structure may impact the peptide–microorganism interaction, leading to a higher peptide concentration required to obtain the same effect. It might also explain some differences found between our research and others demonstrating FbPA's antimicrobial activity (Tang et al., 2002).

The synthetic FbPA exhibited the strongest antifungal activity against *C. tropicalis* at 5.2–10.5 μM in poor medium. Other fungi were incapable of growth after 72 h of incubation in either rich or poor medium at a higher peptide concentration (42–84 μM). A common recurrent infection that affects general insect species is induced by filamentous fungi and is described in several wild insects (Pagnocca et al., 2011; Biedermann et al., 2013; Bateman et al., 2015; Moubasher et al., 2017). These results indicate resistance specificity of the peptide against these microorganisms, suggesting that FbPA might play a role to help increase the efficiency of the insect's immunological barrier when facing these infections.

The inhibition assay of the FITC–FbPA conjugate indicates that it was active against *M. luteus* at a 0.02 mg/mL concentration. Although a higher concentration was necessary for it to be active, this result corroborates previous inhibition results: the native FbPA was active against the same *M. luteus* strain at 0.002 mg/mL and the synthetic variant at 0.2 mg/mL. This change might occur due to the coupling of the FITC to the peptide via its amino groups. As the sequence has three possible FITC binding sites, it could be covering one of the major active sites of the molecule, thus interfering with its action against the bacteria.

In humans, the cleavage of fibrinogen chains is via thrombin action. Thrombin cleaves a specific Arg-Gly at the C-terminus during the last part of the clot formation pathway, resulting in the release of FbPA and B (Riedel et al., 2011). Similarly, invertebrates produce molecules called fibrinogen-related peptides (FREP). Components of this class have been identified in ascidians, echinoderms, annelids, arthropods, nematodes, cnidarians, and molluscs (Wang et al., 2005; Fan et al., 2008; Sterba et al., 2011; Chai et al., 2012). Their function is related mainly to defense mechanisms such as agglutination and antimicrobial action (Hanington and Zhang, 2011). Although some similarities to specific portions of human fibrinogen have been identified, there are no known similarities between FREPs and human FbPA. Considered together with the fact that the *T. infestans* feeds on human blood, our results suggest that this insect can assimilate FbPA during feeding and internalize it in the midgut. In turn, it may use this peptide as an antimicrobial in its haemolymph. Similar strategies have already been described in different invertebrates (e.g., Fogaça et al., 1999; Riciluca et al., 2012). Beyond this capacity, some insects can absorb whole or partial molecules obtained during feeding (Jeffers and Roe, 2008). The presence of this internalization capability has been observed in *Rhodnius prolixus* (Wigglesworth, 1943), phylogenetically related to *T. infestans* that assimilates human hemoglobin.

The introduction of the insect proboscis into the host tissue causes local damage that activates immune responses as well as the clotting cascade, leading to thrombin activation and FbPA production (Scheraga, 2004). This pathway, however, is inhibited due to the release of thrombin inhibitors expressed within insect saliva (Zavalova et al., 2002). The presence of an anticoagulant buffer in the blood offered to the insects suggests that the cleavage can occur in the midgut, but this piece of information isolated does not represent a definite answer to this issue, because we analyzed only the blood with the presence of citrate buffer. It would be necessary to analyse the haemolymph of *T. infestans* after it feeds on a real organism instead of an *in vitro* system. Thus, it remains to be investigated whether FbPA internalized by *T. infestans* comes from endogenous cleavage within the host or another process.

Moreover, the role played by the peptide in the *T. infestans* haemolymph *in vivo* requires further investigation. Finally, further research is warranted as to the peptide's local action and its potential application as a therapeutic agent against infectious diseases.

Therefore, our results demonstrate the presence of antimicrobial active human FbPA in the haemolymph of the blood-sucking insect *T. infestans*. The hypothesis confirmed was that the presence of this molecule on the *T. infestans* haemolymph happens through intestinal absorption, through FITC–FbPA internalization experiments.

This discovery allows us to confirm that blood-sucking insects can gather different molecules from various resources as an attempt to defend themselves against pathogens. These results also contribute to a wider comprehension of the insect immune system, such as its role on an evolutive scale, and the results generate some necessary information to facilitate the discovery of new sources of antimicrobial peptides.

## Author contributions

LD was mainly responsible for the development of all experiments and writing of the manuscript. PS participated during the purification experiments, mainly HPLC, and all of the internalization experiments. AM participated during the solid-phase synthesis of the peptide and mass spectrometry. All the authors contributed to the manuscript and development, and the entire group approves the entire manuscript content.

### Conflict of interest statement

The authors declare that the research was conducted in the absence of any commercial or financial relationships that could be construed as a potential conflict of interest.
